# An *in vitro* analysis of how lactose modifies the gut microbiota structure and function of adults in a donor-independent manner

**DOI:** 10.3389/fnut.2022.1040744

**Published:** 2023-01-26

**Authors:** Jenni Firrman, LinShu Liu, Karley Mahalak, Weiming Hu, Kyle Bittinger, Ahmed Moustafa, Steven M. Jones, Adrienne Narrowe, Peggy Tomasula

**Affiliations:** ^1^Dairy and Functional Foods Research Unit, Eastern Regional Research Center, Agricultural Research Service, U.S. Department of Agriculture, Wyndmoor, PA, United States; ^2^Division of Gastroenterology, Hepatology, and Nutrition, The Children’s Hospital of Philadelphia, Philadelphia, PA, United States; ^3^Department of Pediatrics, Perelman School of Medicine, University of Pennsylvania, Philadelphia, PA, United States

**Keywords:** lactose-intolerance, milk, gut microbiota, lactic acid bacteria (LAB), *Bifidobacterium*, lactate

## Abstract

**Introduction:**

Following consumption of milk, lactose, a disaccharide of glucose and galactose, is hydrolyzed and absorbed in the upper gastrointestinal tract. However, hydrolysis and absorption are not always absolute, and some lactose will enter the colon where the gut microbiota is able to hydrolyze lactose and produce metabolic byproducts.

**Methods:**

Here, the impact of lactose on the gut microbiota of healthy adults was examined, using a short-term, *in vitro* strategy where fecal samples harvested from 18 donors were cultured anaerobically with and without lactose. The data were compiled to identify donor-independent responses to lactose treatment.

**Results and discussion:**

Metagenomic sequencing found that the addition of lactose decreased richness and evenness, while enhancing prevalence of the β-galactosidase gene. Taxonomically, lactose treatment decreased relative abundance of *Bacteroidaceae* and increased lactic acid bacteria, *Lactobacillaceae, Enterococcaceae*, and *Streptococcaceae*, and the probiotic *Bifidobacterium*. This corresponded with an increased abundance of the lactate utilizers, *Veillonellaceae*. These structural changes coincided with increased total short-chain fatty acids (SCFAs), specifically acetate, and lactate. These results demonstrated that lactose could mediate the gut microbiota of healthy adults in a donor-independent manner, consistent with other described prebiotics, and provided insight into how dietary milk consumption may promote human health through modifications of the gut microbiome.

## Introduction

One major pathway by which dietary constituents can affect changes in human health is through the gut microbiota, which is a large community of microorganisms that reside throughout the gastrointestinal tract (GIT) (1). Although it contains an array of microbial types (2), the bacterial community is the dominant subject of research because it is known to play a key role in digestion, immune protection, synthesis of vitamins and other bioactive molecules, and the release of metabolic byproducts including short-chain fatty acids (SCFAs) (3). Research on this active community has found that diet is a central factor that dictates diversity and metabolic activities and can modify community structure in a harmful or healthy manner (2, 3). There have been a number of studies looking at how diet types, i.e., the western diet and plant-based or vegan diet, or diets high in fat and sugar, animal protein, or dietary fiber, can affect the gut microbiota in terms of temporal changes (4–7). Interestingly, although bovine milk and dairy products are consumed worldwide, research on how this food group may affect changes to the adult gut microbiota is comparatively limited (8).

Consumption of bovine milk or dairy products is recommended as part of a healthy diet for all age groups (9, 10), because it is a dense source of multiple required nutrients such as proteins, fat, carbohydrates, minerals, vitamins, and other trace elements (11, 12). Physiologically, the consumption of bovine milk has been associated with a number of health benefits including antioxidant effects, prevention of osteoporosis, reduced risk of childhood obesity and type 2 diabetes, protection against the development of some types of cancer, and is inversely associated with hypertension, stroke, and other cardiometabolic diseases (10, 13–15). A large proportion of the data generated used to report these health benefits come from human studies or meta-analyses, looking at the consumption of milk or dairy products and correlating this with the health outcome. However, there is ongoing research attempting to elucidate which components of bovine milk are responsible for the desired effects, and their mechanism of action.

Lactose is a unique component of mammal milk, present at a concentration of approximately 4.6 g/100 mL in bovine milk (16). Chemically, it is a disaccharide of glucose and galactose linked by a β-1-4 glycosidic bond between the carbon 4 of glucose and carbon 1 of galactose (16, 17). Under normal circumstances, following consumption, it is hydrolyzed by the enzyme lactase (β-galactosidase) in the proximal small intestine into the monosaccharides glucose and galactose which are then absorbed (16–18). However, it has been found that not all lactose is metabolized and absorbed in the small intestine, and some dietary lactose may enter the colon (19). The amount of lactose that reaches the colon is variable and based on how much is consumed in the diet coupled with the genetic background of the individual, as most people are lactase non-persistent, and therefore are lactase deficient and unable to effectively hydrolyze lactose in the small intestine (1, 16, 17, 19, 20).

In the colon, a large proportion of gut microbes have the genetic capacity to produce their own β-galactosidase enzyme and are therefore able to hydrolyze lactose, utilize the resulting monosaccharides, and produce byproducts such as lactate, short-chain fatty acids (SCFAs), H_2_, CO_2_, and CH_4_ (21, 22). Based on this information, it is a reasonable hypothesis that the consumption of lactose may impact the composition and metabolome of the gut microbiota. In fact, the results of a few recent studies have indicated that lactose is a healthy modifier of the gut microbiota, functioning as a prebiotic (22–24). Previous human studies looking at lactose consumption and the gut microbiota using fecal samples of adults, have found a positive correlation between lactose and the presence of *Bifidobacterium* (25), specifically *B. adolescentis, B. longum, B. bifidum*, and the levels of these taxa further increased for individuals who were lactase non-persistent yet still consumed lactose or dairy products (26). In studies looking at the infant gut microbiota *in vitro*, lactose treatment was found to increase the abundance of *Bifidobacterium* and *Lactobacillus*, decreased the abundance of pathogens, and enhanced levels of acetate and lactate (27, 28).

The current study expanded on these previous findings, with the specific goal of analyzing the effect of lactose on the fecal microbiota of healthy adults. To examine the direct effect of lactose on the microbial community an *in vitro* design was utilized, which eliminated interference from mammalian components present in an *in vivo* model, such as host produced enzymes and immune factors. Fecal samples from 18 adult donors between the ages of 25–70 were incubated in cultures anaerobically for 24 h, with and without lactose. Metagenomic sequencing, targeted sequencing using qPCR, and metabolic profiling were applied to elucidate the effect of this milk component on community structure and function. Furthermore, the combined analysis of data generated from all 18 donors allowed for the identification of donor-independent responses to lactose in terms of both structure and function. Utilization of an *in vitro* system specifically to study the effect of lactose on the adult gut microbiota provided novel results and insight into how lactose may contribute to the overall health benefits of drinking milk for the adult population.

## Materials and methods

### *In vitro* culturing experiments

Fresh feces were harvested from 18 random adult donors between the ages of 25–70 years old. Donors were excluded if they had any GI disorders, including cancer, were taking any medication to treat psychosis or allergies, or were pregnant or lactating. All donors were non-smokers, that consumed less than 3 alcoholic beverages on a daily basis, presented with a BMI <30, and had not taken any antibiotics, prebiotics, or probiotics for at least 3 months. Each donor had a unique microbial community as determined by 16S rRNA gene sequencing (Supplementary Figure 1). Donors provided informed consent and IRB approval was received prior to fecal collection. Fresh feces from each donor (7.5 g) were homogenized anaerobically in 100 mL of phosphate buffer containing 8.8 g/L K_2_HPO_4_; 6.8 g/L KH_2_PO_4_; 0.1 g/L 87 sodium thioglycolate; and 0.015 g/L sodium dithionite to create a 7.5% fecal slurry as described previously (29, 30). The fecal slurry was used to inoculate two anaerobic culture tubes at a 10% final volume, one containing anaerobically prepared nutritional media only (no treatment), and one containing nutritional media supplemented with 5 g/L of α-Lactose monohydrate (Carl Roth) (lactose treated) (Supplementary Figure 2). The dosage was selected because there is approximately 5 g/100 mL of lactose in milk (31), the USDA recommends adults consume 3 servings of dairy/day (9) and it has been reported that 0–8% of lactose was unabsorbed in the ileum of lactose tolerant and 42–75% unabsorbed in mildly intolerant individuals (19). The dose used here would be considered a high dose, which was selected to ensure that any effects that occurred would be measurable. Additionally, the control was media only, so the lactose treated group received a larger source of carbon compared to the control group. The anaerobic culture tubes were sealed to ensure anaerobiosis. The basal nutritional media was pH 6.5 and contained the following commercially available ingredients: 16.3 g/L KH_2_PO_4_, 5.2 g/L K_2_HPO_4_, 2.0 g/L Yeast Extract, 2.0 g/L peptone, 2.0 g/L NaHCO_3_, 2.0 mL/L Tween80, 1.0 g/L mucin, 0.5 g/L L-cysteine. The inoculated anaerobic culture tubes were incubated at 37°C for 24 h and samples harvested from each donor at time 0 (inoculum) and from both the no treatment and lactose- treated groups at 6- and 24-h post-inoculations for metagenomic and functional analysis.

### pH measurements and functional analysis

During the experiment, pH was monitored for each anaerobic culture tube using a Senseline pH meter F410 (ProSense, Oosterhout, Netherlands). Samples were harvested as described above and used to quantify the amounts of short chain fatty acids (SCFA) and lactate. SCFAs were extracted with diethyl ether and analyzed using a GC-2014 gas chromatography (Shimadzu) instrument as described previously using a 1 μL injection volume (32). A commercially available kit (R-Biopharm, Darmstadt, Germany) was used to determine lactate levels following the manufacturer’s guidelines. SCFAs detected were acetate, propionate, butyrate, valerate, and branched chain SCFAs (BCSCFAs), isobutyrate, isovalerate, and isocaproate. Total SCFAs were calculated as the sum of all SCFAs listed.

### qPCR analysis for *Bifidobacterium* and total bacteria quantification

Deoxyribonucleic acid was extracted from a 1 mL volume using a fast DNA spin kit for soil (MP Biomedical) and eluted to a final volume of 100 μL. A qPCR assay was used to determine levels of *Bifidobacterium* genus and total bacteria as described previously (33, 34). Primers and G-blocks (synthesized gene fragments) used for standards were ordered from Integrated DNA Technologies (IDT) and reconstituted following the manufacturer’s guidelines. Standards were run using 10× serial dilutions from 1 × 10^7^ to 1 × 10^2^ copies/μL. Extracted DNA from each sample was diluted 100× in qPCR grade water (Roche). All samples were run in triplicate, and any sample that fell outside of the range of the standard were further diluted. For *Bifidobacterium* genus the primers were the following: forward Bif243F 5′-TCGCGTCYGGTGTGAAAG-3′ and reverse Bif243R 5′-CCACATCCAGCRTCCAC-3′ (33). For total bacteria the primers targeted the V3-V4 16S gene region and were the following: forward 338F 5′-ACTCCTACGGGAGGCAGCAG-3′ and reverse 518R 5′-ATTACCGCGGCTGCTGG-3′ (34). For both assays, the total reaction volume was 20 μl, containing 1 μl diluted DNA, 500 nM forward primer, 500 nM reverse primer, and 2× Applied Biosystems SYBR Green PCR Master Mix (Fisher Scientific, Waltham, MA, USA).

The negative controls of no DNA, no forward primer, no reverse primer, and water only were included in each plate. For *Bifidobacterium* genus the following times and temperatures were used for the reaction: 95°C for 5 m, followed by 40 cycles of 95°C for 15 s, 58°C for 20 s, and 72°C for 30 s, and ended with 83°C for 30 s, 94°C for 15 s and a melting curve analysis. For total bacteria the following times and temperatures were used for the reaction: 95°C for 5 m, followed by 40 cycles of 95°C for 15 s, 64°C for 15 s, and 72°C for 30 s, and ended with 83°C for 15 s and a melting curve analysis. Absolute quantification of bacterial cells per μL of DNA was calculated using the Roche Lightcycler software. Outliers were identified by an Interquartile Rule test and removed from the analysis. Results are portrayed as the average of all donors, with standard deviation. Statistical differences between the no treatment versus the lactose treated group were performed using a student’s *t*-test and considered significant if *P* < 0.05.

### DNA sequencing and processing

Barcoded PCR primers annealing to the V1-V2 region of the 16S rRNA gene were used for library generation. PCR reactions were carried out in duplicate using the Q5 High-Fidelity DNA Polymerase (New England Biolabs). PCR reactions contained 0.5 uM of each primer, 0.34 U Q5 Pol, 1× Buffer, 0.2 mM dNTPs, and 2.5 ul DNA in a total volume of 25 ul. Cycling conditions were as follows: 1 cycle of 98°C for 1 m; 25 cycles of 98°C for 10 s, 56°C for 20 S, and 72°C for 20 s; 1 cycle of 72°C for 8 m. After amplification, PCR reactions were pooled and purified using a 1:1 volume of SPRI beads. DNA in each sample was quantified using PicoGreen and pooled in equal molar amounts. The resulting library was sequenced on the Illumina MiSeq using 2 × 250 bp chemistry. Extraction blanks and DNA-free water were subjected to the same amplification and purification procedure to allow for empirical assessment of environmental and reagent contamination. Positive controls, consisting of eight artificial 16S gene fragments synthesized in gene blocks and combined in known abundances were also included. Sequence data were processed using QIIME2 v2019.7 (35). Using QIIME2 v2019.7 plugins, read pairs were processed to identify amplicon sequence variants with DADA2 (36). Taxonomic assignments were generated by comparison to the Greengenes reference database (37), using the naive Bayes classifier implemented in scikit-bio (38). A phylogenetic tree was inferred from the sequence data using MAFFT (39). Similarity between samples was assessed by weighted and unweighted UniFrac distance (40, 41), as well as percent shared species (Jaccard index) and Bray-Curtis distance.

### Bioinformatic and statistical analysis

Data files from QIIME were analyzed in the R environment for statistical computing. Global differences in bacterial community composition were visualized using Principal Coordinates Analysis. Sample groups were compared at the community level using the PERMANOVA test (42). The relative abundance of bacterial taxa was compared using linear mixed effects models after log transformation.

### Pearson’s correlation and PICRUSt2 analysis

To examine correlation between taxa and SCFAs and lactate, Pearson correlations were calculated between each byproduct and each taxon with taxon relative abundance summed at the genus level. Multiple testing correction was applied using the Benjamini and Hochberg method using the R stats:p.adjust function. For visualization, correlations were filtered to include the taxa identified as differentially abundant and shown in Figure 5, and taxa having significant correlations to any of the SCFAs or lactate. These correlations were plotted using the R corrplot package and labels were edited using Inkscape v 1.1^1^ (43). PICRUSt2 v2.4.1 was used to infer microbial community genomic functions and estimated counts of the *lacZ* gene (KEGG: K01190) were extracted for further analysis (44). ANOVA (stats::aov) in R v 4.1.3 was used to test for differences in *lacZ* gene prevalence by treatment, by age group, and by time point.

**FIGURE 1:**
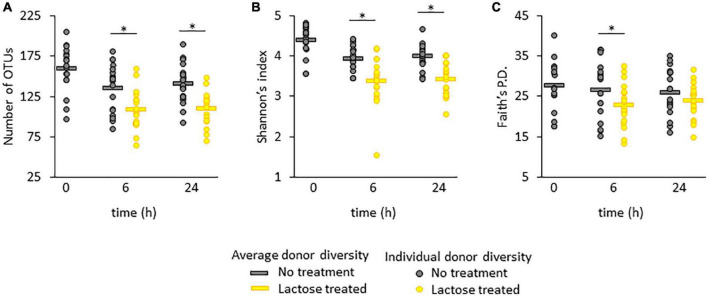
Alpha diversity in terms of **(A)** richness, **(B)** Shannon’s index, and **(C)** Faith’s phylogenetic distance (Faith’s P.D.). Statistical differences were determined using a linear model between the no treatment and lactose treated groups and *P*-values that were < 0.05 are indicated with an asterisk*.

**FIGURE 2:**
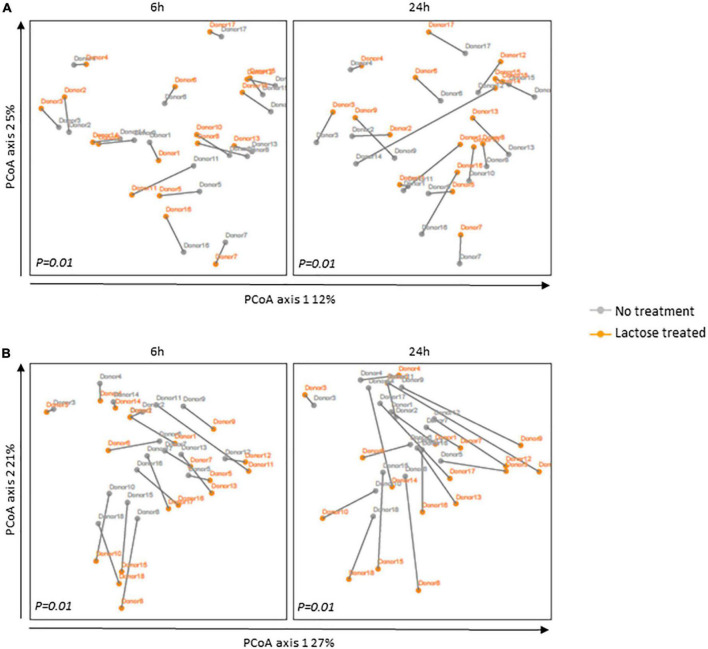
Principal coordinates analysis (PCoA) based on **(A)** unweighted and **(B)** weighted UniFrac distances for the 6- and 24-h timespoints post-inoculation. The communities for each donor are portrayed separately with the gray line indicating their distance. Statistical differences between no treatment and lactose treated groups were determined using PERMANOVA and *P*-values are indicated at the bottom of each box.

**FIGURE 3:**
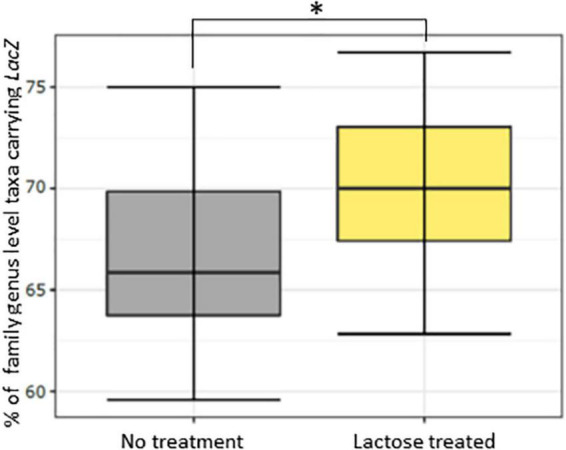
Percentage of family-genus level taxa containing the *LacZ* gene for the no treatment and lactose treated groups as inferred using PICRUSt2. The * asterisk symbol indicates *P* < 0.001.

**FIGURE 4:**
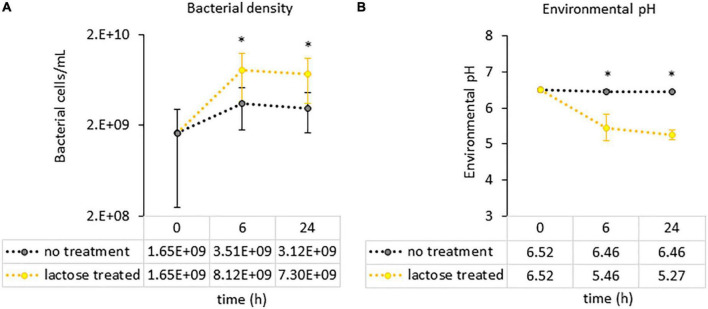
Measurements of culture parameters **(A)** bacterial load and **(B)** environmental pH. Each circle represents the average count with error bars indicating standard deviation. Statistical differences were determined *via* a Student’s *t*-test between the no treatment and lactose treated groups. *P*-values that are < 0.05 are indicated with an asterisk*.

**FIGURE 5:**
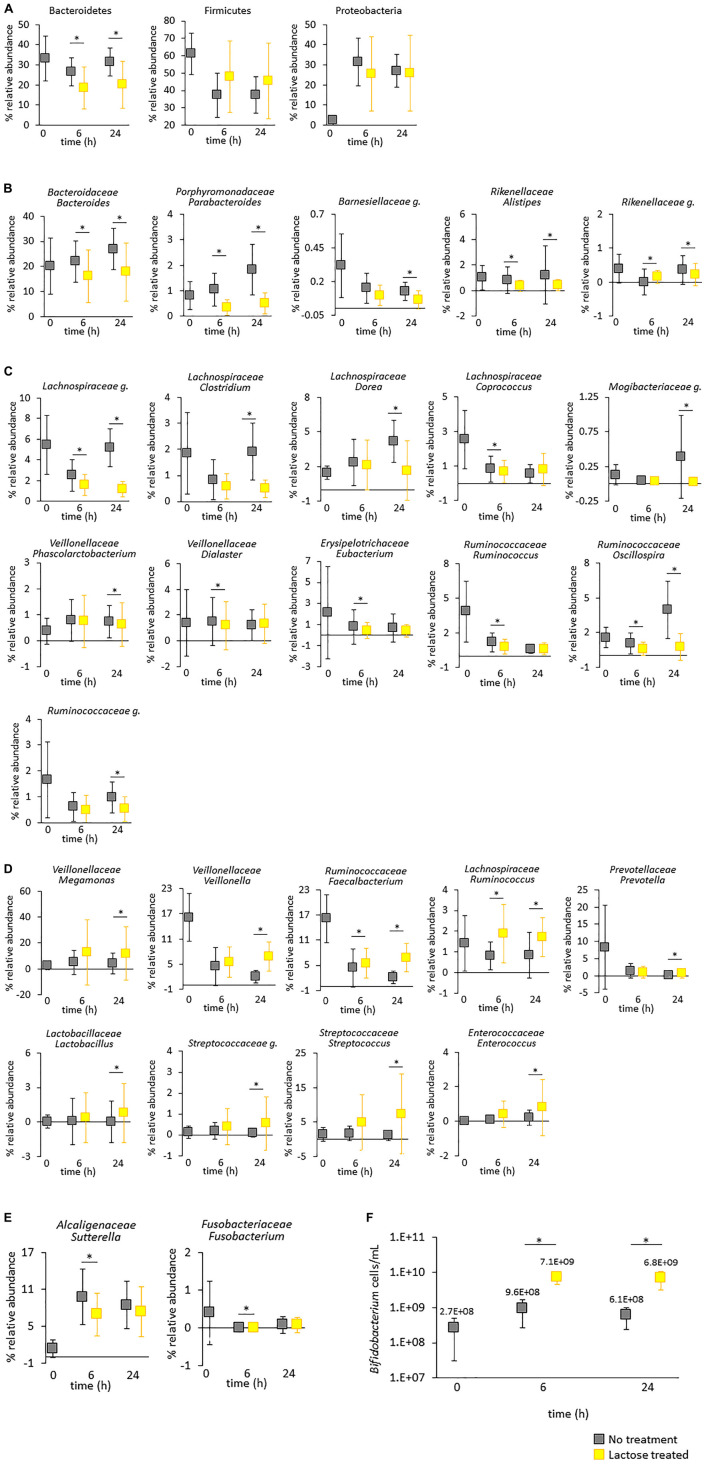
Taxa impacted by lactose treatment. The figures show the average abundance for all donors with standard deviation. **(A–E)** Relative abundance based on 16S rRNA gene sequencing. Statistical differences between the untreated and lactose treated groups was determined using a linear model, adjusted for false discovery rate using the Benjamini–Hochberg method, and are annotated with an asterisk*. **(A)** Phylums Bacteroidetes, Firmicutes, and Proteobacteria. **(B)** Taxa within phylum Bacteroidetes. **(C)** Taxa within phylum Firmicutes that increased and **(D)** decreased due to lactose treatment. **(E)** Taxa within phylums Proteobacteria and Fusobacteria. **(F)** Levels of *Bifidobacterium* genus were quantified *via* qPCR. Statistical differences between the no treatment and lactose–treated groups were determined *via* a Student’s *t*-test. *P*-values that are < 0.05 are indicated with an asterisk*.

## Results and discussion

### Lactose treatment decreased alpha diversity and shifted the microbial community structure to favor taxa with the genetic capacity to produce the β-galactosidase enzyme

The goal of this study was to analyze the effect of lactose on the adult gut microbiota, and to identify donor-independent effects that may occur. To achieve this goal, a small volume, batch-culture method was employed which allowed for higher-throughput compared to the larger, multi-vessel *in vitro* systems. This high-throughput design only permitted a short-term experiment (24 h), and the application of a single dose of lactose. However, the benefit of this *in vitro* experimental design is that it allowed for the independent analysis of 18 adult donor samples and provided a wealth of information on how these different communities were influenced by lactose, in both divergent and convergent manners.

To begin, fecal samples collected from 18 adult donors were cultured in sealed, anaerobic culture tubes without (no treatment group) and with lactose (lactose treated group) over a 24-h period. Samples were harvested prior to cultivation at time 0, and from both groups at 6- and 24-h post-inoculation. DNA was extracted for 16S rRNA gene sequencing of the V1-V2 region using the Illumina Miseq platform. Alpha diversity for each sample was calculated in terms of richness, Shannon’s diversity, and Faith’s phylogenetic diversity (Faith’s P.D.). The results from all donors in either the no treatment or lactose treated groups were averaged together and statistical analysis was used to identify donor-independent changes.

For all three metrics, the average measurement was highest at time 0 and decreased at the 6- and 24-h time points for both the no treatment and lactose treated groups (Figures 1A–C). For richness and Shannon’s diversity, the presence of lactose significantly reduced these amounts compared to the control at both the 6- and 24-h timespoints (Figures 1A, B), whereas Faith’s P.D. was only significantly lowered by the presence of lactose at the 6-h timepoint (Figure 1C). These results indicated that lactose was effectively altering community structure; specifically, the decrease in richness, or the number of detectable taxa, coupled with the reduction in Shannon’s diversity, or community evenness, indicated that lactose treatment favored the outgrowth of a few, select taxa.

The changes in alpha diversity corresponded with a shift in community structure, as illustrated by a principal coordinate analysis based on UniFrac distances (Figure 2). Although the donor communities were variable from each other, in both the unweighted and weighted analysis, there is a clear separation between each donor’s community in the no treatment versus lactose treated group at both the 6- and 24-h time points which achieved statistical significance. The unweighted analysis depicted changes that occurred simply from the presence or absence of taxa and the separation between the no treatment and lactose treated groups showed that the addition of lactose was driving the abundance of some taxa above or below the threshold of detection (Figure 2A). Expanding further, the weighted analysis showed that there were not only changes in the presence or absence, but also in the abundance of these taxa within each community (Figure 2B).

The PCoA results of both unweighted and weighted UniFrac distances correlated to the observed decrease in richness and Shannon’s diversity (Figure 1), further evidencing that the addition of lactose shifted community structure in terms of composition and abundance of the dominant taxa. These data suggested that lactose treatment selected for taxa capable of expressing the β-galactosidase enzyme and able to metabolize this compound, thereby providing a nutritional, competitive advantage. This hypothesis was supported by the results of a previous study on *Escherichia coli* which found that the presence of the *lac* operon, which carries the β-galactosidase gene, provided a competitive advantage for this taxon to colonize the intestines of mice when lactose was supplied as a nutrient source (21). Additionally, it was previously reported that lactase non-persistent humans consuming dietary lactose had increased fecal β-galactosidase activity and lowered fecal pH, which pointed toward an increase in acid-tolerant, lactose utilizing taxa, such as lactic acid bacteria *Lactobacillus*, and *Bifidobacterium* (22, 45, 46).

To see if the results of this study aligned with these previous reports, a PICRUSt2 analysis was used to evaluate genetic capacity to metabolize lactose by looking at the prevalence of the *LacZ* gene, which is the portion of the Lac operon that genetically codes for the β-galactosidase enzyme (47). The results of the PICRUSt2 analysis found that the lactose-treated communities had a significantly higher percentage of taxa carrying the *LacZ* gene at the family-genus level (Figure 3). Furthermore, environmental pH was significantly reduced in the lactose treated group while community density was increased at both the 6- and 24-h timespoints (Figures 4A, B). These results aligned with the previous findings and, together, provided evidence that the addition of lactose selected for taxa able to utilize it as a carbon source and produce acidic byproducts. The corresponding drop in pH that occurred due to the metabolism of lactose can be considered as a limitation of this study design, as it has been previously shown that decreasing environmental pH can affect the gut microbiota structure and function (51).

### Lactose affects taxonomic composition of the gut microbiota community by decreasing Bacteroidetes and enhancing levels of lactic acid bacteria taxa and the probiotic *Bifidobacterium*

The initial results indicated that lactose modified community structure by reducing richness and evenness and selecting for taxa carrying the *LacZ* gene. To further explore the structural dynamics of the gut microbiota in response to lactose, results of the 16S rRNA sequencing were used to generate a profile of taxa statistically affected in a donor-independent manner (Figure 5). Of the three most dominant phyla, there was a statistically significant decrease in Bacteroidetes (*P* < 0.05) due to lactose treatment that occurred at both the 6- and 24-h time points, but this was not the case for Firmicutes or Proteobacteria due to the large variations between donors (Figure 5A). Interestingly, all the identified statistically significant changes that occurred within Bacteroidetes were decreases to relative abundance; no taxa within Bacteroidetes increased in a statistically significant manner in response to lactose.

The decrease in Bacteroidetes was primarily due to *Bacteroides*, which was the dominant genus within this phylum and significantly reduced at both the 6- and 24-h time points (Figure 5A). *Bacteroides* species are well known for their polysaccharide utilization systems (PULs) that produce an extensive repertoire of enzymes that degrade polysaccharides and produce oligosaccharides that serve as carbon sources for surrounding bacteria in multifactorial, cross-feeding interactions (48). A number of taxa within *Bacteroides* also carry β-galactosidase genes and are capable of hydrolyzing lactose, yet in this study the relative abundance of this genus decreased in response to lactose treatment (49). It has been previously reported that *Bifidobacterium longum* is more efficient at importing simple sugars compared to *Bacteroides thetaiotaomicron*, which suggested that the decrease in *Bacteroides* observed here may not be due to an inability to utilize lactose as a carbon source, but to outcompete fellow lactose-utilizing taxa within the community due to other selective pressures such as environmental pH (54). Previously, it has been reported that a decrease to environmental pH resulted in a decrease to levels of Bacteroidetes (50). Here, it is possible that the observed significant decrease for *Parabacteroides, Alistipes* and the unidentified genus within family *Rikenellaceae* (*Rikenellaceae g.)* at the 6- and 24-h timespoints was due to the decrease in environmental pH, as these taxa have been previously shown to be reduced in acidic conditions (51).

Unlike Bacteroidetes, Firmicutes did not significantly respond to lactose at the phylum level; However, there were several taxa within this phylum that were lactose responsive. A total of 11 taxa within Firmicutes decreased in relative abundance due to lactose treatment, with the largest number of taxa coming from family *Lachnospiraceae* followed by *Ruminococcaceae* (Figure 5C). It is difficult to hypothesize why the specific genera within these families were affected in this experiment due to community complexity, especially since family *Lachnospiraceae* is a diverse class within the Clostridium Cluster XIVa that is described as having large inter- and intra- species diversity (52). *Oscillospira*, which is part of family *Ruminococcaceae*, are thought to degrade host glycans or rely on interspecies cross-feeding from taxa such as *Bacteroides sp.* as their primary source of nutrition (53). In this case, the decrease in *Oscillospira* may be linked to the decrease in *Bacteroides* and the increase in metabolic byproducts of lactose. Alternately, the decrease to pH may have effected taxa within *Lachnospiraceae* and *Ruminococcaceae* following results from a previous report (51). There were a few taxa that were only decreased at the 6-h timepoint, which may be due to growth kinetics since they were no longer significant at the 24-h timepoint.

There were 9 taxa within phylum Firmicutes that increased in response to lactose treatment (Figure 5D). Most notably, at the 24-h timepoint there was an increase in several taxa classified as lactic acid bacteria (LAB), specifically *Lactobacillus*, an unidentified genus of *Streptococcaceae* (*Streptococcaceae g.*), *Streptococcus*, and *Enterococcus*, and a corresponding increase in lactate utilizers, *Veillonella* and *Megamonas*. LABs are often used for food fermentation or probiotics and are characterized as converting carbohydrate sources into lactate, although they also produce an array of other beneficial metabolites as well (54, 55). *Veillonellaceae* is a gram-negative taxon within Firmicutes that is well-known to ferment lactate and produce acetate, propionate, and CO_2_ (56–58). These results suggest a cooperative interaction where lactose is converted to lactate, which is then utilized by the lactate consumers, and supports the previous supposition that lactose consumption may lead to an increase in LAB taxa, lactate utilizers, and *Bifidobacterium* (22, 45, 46). The data here also suggested that this interaction may extend beyond the colon to the small intestine, as families *Lactobacillaceae, Streptococcaceae, Veillonellaceae, Enterococcaceae*, and *Bifidobacterium* are common members of the small intestine gut microbiota as well as the colon (55, 59).

There were no statistical changes at the phylum level to Proteobacteria and Fusobacteria, however, within each of these phyla one taxon was significantly impacted. However, these changes were quite minimal and only at the 6-h timepoint (Figure 5E). One genus that was missing from the 16S analysis was *Bifidobacterium*, which has been previously shown to increase due to the presence of lactose and can produce and utilize lactate (56). However, it has been previously found that sequencing of the V1-V2 region of the 16S gene using traditional V1-V2 primers does not accurately detect this taxon (60). To address this, a qPCR assay was used to quantify levels of *Bifidobacterium* within each sample and found that levels of *Bifidobacterium* were significantly and drastically increased due to lactose treatment at both the 6- and 24-h timespoints (Figure 5F). Compared to the control, in the lactose treated group the levels of *Bifidobacterium* were 7.4 times higher at the 6-h timepoint and 11.2 times higher at the 24-h timepoint. These results aligned with similar reports that showed lactose enhanced levels of *Bifidobacterium* in human subjects, and these levels were further enhanced in people who are lactase non-persistent, yet still consume lactose (25, 26).

Together, the results from metagenomic sequencing and qPCR provided evidence that lactose was altering community structure in a donor-independent manner. These findings are important because it has been previously determined that the structure of the gut microbiota is predictive of its function, i.e., the taxa that comprise the community are responsible for the metabolic output (61). Therefore, whether or not the addition of lactose altered community functionality was assessed next.

### Lactose driven modifications to the community structure corresponded with changes to metabolic output

**FIGURE 6:**
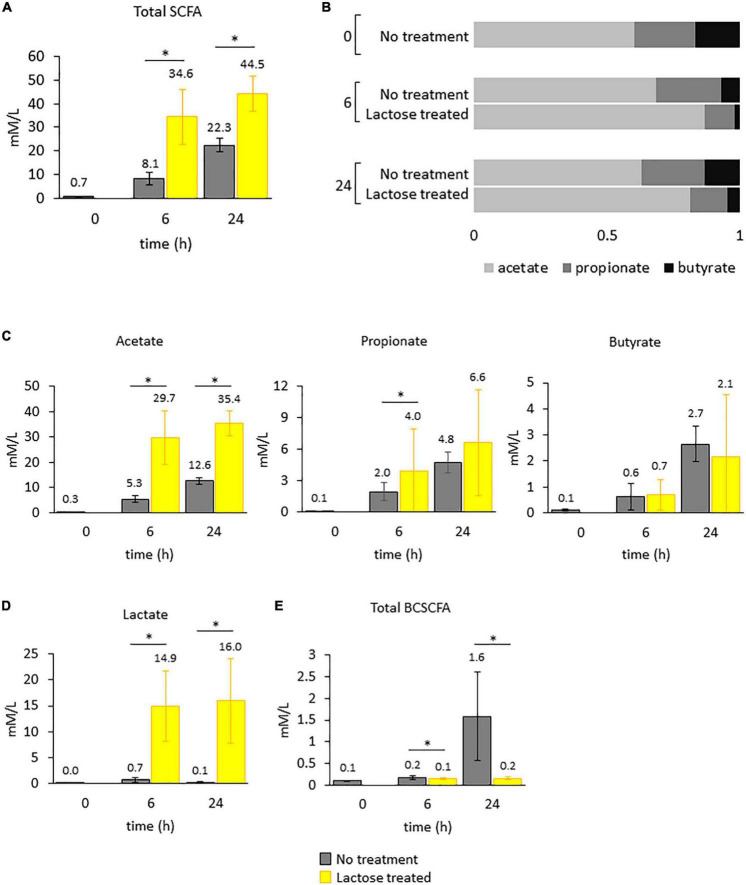
Levels of metabolic products were quantified for each donor over time. Bars in the figure represent the average amounts detected with standard deviation. Statistical differences were determined *via* a Student’s *t*-test between the no treatment and lactose treated groups. *P*-values that are < 0.05 are indicated with an asterisk*. **(A)** Total SCFAs. **(B)** Ratios of acetate to propionate to butyrate. **(C)** Acetate, propionate, and butyrate. **(D)** Lactate. **(E)** Total BCSCFA.

Within the GIT, the gut microbiota is functionally active and ferments non-digestible substrates into the end-product metabolites termed short-chain fatty acids (SCFAs), predominantly acetate, propionate, and butyrate. These can be used by the other microbes within the community or by the mammalian cells and are positively associated with gut health (62). To determine if the lactose-driven changes to community structure would affect functional output, levels of SCFAs and lactate were quantified, and the results from all donors were combined to identify a donor-independent functional response to lactose (Figure 6).

In the lactose treated group, total SCFAs were increased at both the 6- and 24-h timepoints (Figure 6A). This enhanced metabolic output was expected, since the lactose-treated group was provided an additional source of carbon and had increased cell density and lowered environmental pH (Figure 4). However, the ratio of acetate to propionate to butyrate for the lactose-treated group was altered compared to the non-treated group at both timepoints, a metric that was unaffected by cell density (Figure 6B). Acetate was the primary contributor to the increased levels of SCFAs in response to lactose, and was significantly higher at both timepoints, followed by propionate, which was significantly increased only at the 6-h timepoint, while butyrate levels remained consistent between both groups (Figure 6C). In addition to SCFAs, levels of lactate were exponentially increased in the lactose-treated group at both timepoints (Figure 6C). These results showed that lactose was being converted primarily into acetate and lactate in a donor-independent manner.

Compared to the lactose-treated group, levels of branched-chain SCFAs (BCSCFAs) were significantly higher in the no treatment group (Figure 6C). BCSCFAs are produced from the fermentation of amino acids (62). These results suggest that in the no treatment group, metabolism of the carbohydrate and protein sources occurred over time, evidenced by the release of both SCFAs and BCSCFAs. Conversely, the lactose treated group, which was provided an extra carbon source, failed to produce BCSCFAs to the same extent. This could mean that the fermentation of the provided carbohydrates was favored over amino acids, and by 24 h post-inoculation the no treatment group had utilized the carbohydrate sources and switched to protein metabolism where the lactose treated group had an excess of carbohydrates; however, this is speculative since metabolomics to determine the content of the samples at the end of the experiment was not performed.

It should be noted that although some of the observed changes to community structure in this study, i.e., the decrease in *Bacteroidetes, Lachnospiraceae*, and *Ruminococcaceae*, are consistent with changes previously found to occur due to more acidic culturing conditions, the observed increase in acetate and propionate, and decrease in butyrate are not (51). In fact, a previous report found that lowering environmental pH significantly decreased total SCFA levels, and specifically decreased acetate and propionate, while increasing butyrate (51). Based on this information, it is proposed that the acidic conditions created by lactose supplementation may have influenced community structure, it was not the main driver of the observed changed in functional output. We would hypothesize that the main driver would be the addition of lactose and subsequent production of lactate.

Together, these functional results aligned with the observed changes to community structure. There was a significant increase in taxa that produce lactate, such as the LAB strains and *Bifidobacterium*, which corresponded with increased levels of lactate (Figures 5D, F, 6D). A previous report found that *Bifidobacterium Breve* produced acetate and lactate as the end-products in a carbohydrate excess condition (63); The gut microbiota community is able to convert lactate into acetate, propionate or butyrate (64), and specifically, taxa within *Veillonellaceae* convert lactate into acetate and propionate (56–58). Expectedly, two genera within *Veillonellaceae, Veillonella* and *Megamonas*, and *Bifidobacterium* increased in response to lactose with a corresponding increase in acetate and lactate at both the 6- and 24-h timepoint and propionate at the 6-h timepoint. Additionally, the increase in acetate corresponded with an increase in *Faecalibacterium*, which contains species that are known to convert acetate into butyrate (65, 66).

Interestingly, there was no observed increase in butyrate in this study, even though previous reports have found that lactate can be further converted into butyrate by the gut microbiota (64). Structurally, there was a decrease in genera that contain known butyrate-producing taxa, *Coprococcus* and *Eubacterium*, which would suggest that the decrease in butyrate resulted from a reduction to butyrate-producing taxa. However, *Coprococcus* and *Eubacterium* were only lowered significantly at the 6-h timepoint, and there was an observed increase in *Faecalibacterium* at both the 6- and 24-h timepoints. The decrease in environmental pH was most likely not a factor, since it has been previously demonstrated that butyrate levels are increased in a more acidic environment (51). Based on this information, it is unclear whether butyrate did not increase in this study due to a lack of production, or it’s utilization by members of the community.

**FIGURE 7:**
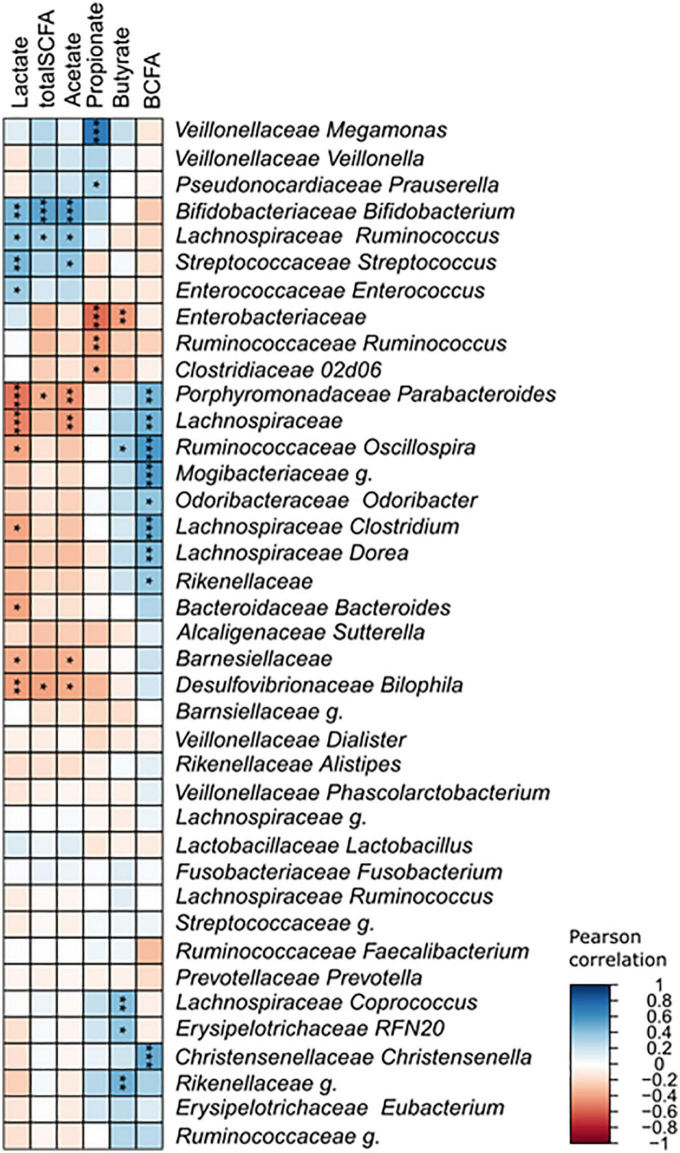
Pearson’s correlation between identified taxa and detected metabolites. Asterisks indicate significant correlations (*p < 0.0; **p < 0.01; ***p < 0.001).

The changes detected in the community structure due to lactose treatment were found to correspond to functional output in a donor-independent manner. However, there was large variation that occurred, shown as a large standard deviation, which stemmed from inter-individual differences of the donors tested. This made it difficult to determine which taxa were correlated to the production of each metabolite. To gain further understanding of the relationship between community structure and function, a Pearson’s correlation was performed that included all samples in the no treatment and lactose-treated groups at the 6- and 24-h timepoints (Figure 7). The results of this analysis clearly showed that the LAB strains, *Streptococcus, Enterococcus*, and *Bifidobacterium* were all significantly, positively correlated with lactate production. This was expected, since it is known that LAB taxa convert lactose into lactate. Alternatively, *Parabacteroides* and an unidentified genus within *Lachnospiraceae* (*Lachnospiraceae g.*) were the most negatively correlated with lactate.

None of the taxa within *Veillonellaceae*, showed any correlation with lactate, which was somewhat unexpected considering that these are known lactate consumers. Reports have found that lactate does not typically accumulate within the GIT because is quickly utilized by the gut microbes (64). One *in vitro* study found that lactate production was independent of environmental pH, but at a low pH lactate utilization was decreased resulting in an accumulation, similar to what was observed here (64). It is possible that the amount of lactate produced in this study was overpowering for the lactate utilizing taxa, or it was produced quicker than it was able to be metabolized.

## Conclusion

It is well-accepted that bovine milk is a healthy source of nutrition and is recommended as a regular part of the diet for all age groups. Compositionally, bovine milk is a mixture of proteins, fats, carbohydrates, vitamins, minerals, and other bioactive compounds. There are several references on the effects of milk proteins on health, but literature on the specific health benefits of lactose are relatively few. Here, the ability of lactose to modify the gut microbiota of 18 adult donors was tested to understand the role it plays in gut microbiome health. The results showed that the addition of lactose led to the reduction of Bacteroidetes and increased levels of LAB taxa and *Bifidobacterium*, and genetic prevalence of the LacZ gene. These structural changes corresponded with an increase in levels of acetate and lactate and enhanced the abundance of lactate utilizing taxa. The increase in acidic byproducts reduced environmental pH, mostly likely contributing to the observed structural changes. These data demonstrated that lactose may be considered a healthy modifier of the gut microbiota community by enhancing beneficial taxa, such as *Bifidobacterium*, and increasing production of healthy metabolites, such as acetate.

## Data availability statement

The data presented in this study are deposited in the NCBI’s Sequence Read Archive, accession number NCBI; PRJNA883645.

## Author contributions

JF, LL, PT, and KM conceptualized the experiment. JF, LL, and KM designed and implemented the experiment. AM and SJ performed the 16S rRNA sequencing and took part in the acquisition and analysis of the sequencing data. KB, WH, and AN performed the bioinformatic analysis. All authors prepared the manuscript.

## Footnotes

1
https://inkscape.org

## Supplementary material

Supplementary Figure 1 Community composition for each donor tested at the family genus level based on 16S rRNA gene sequencing. Only taxa present at a > 1% relative abundance are included.

Supplementary Figure 2 Schematic illustrating the experimental design. *Samples harvested.
