# Supplementary figures and images for: An in vitro analysis of how lactose modifies the gut microbiota structure and function of adults in a donor-independent manner
Source: Front Nutr. 2023 Jan 26;9:1040744. doi: 10.3389/fnut.2022.1040744 (PMC9908759; doi:10.3389/fnut.2022.1040744)

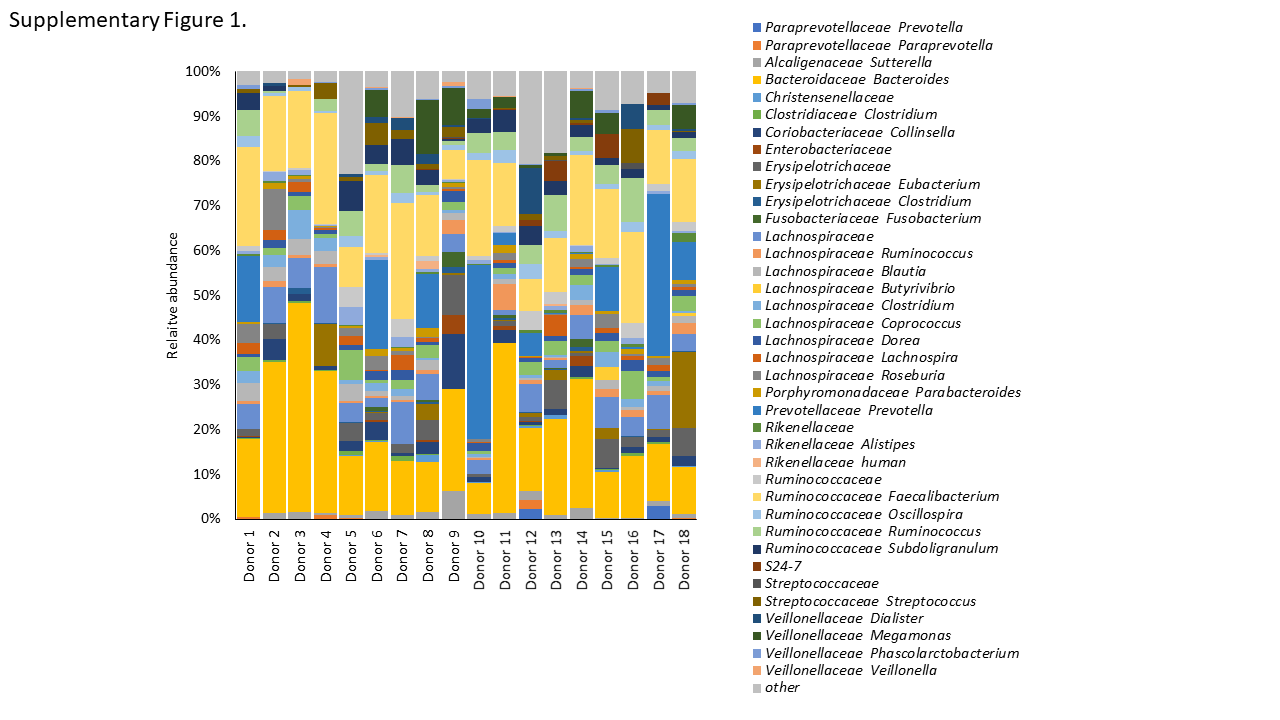

Supplement: Supplementary Figure 1 — Community composition for each donor tested at the family genus level based on 16S rRNA gene sequencing. Only taxa present at a > 1% relative abundance are included. [file Image_1.TIF]

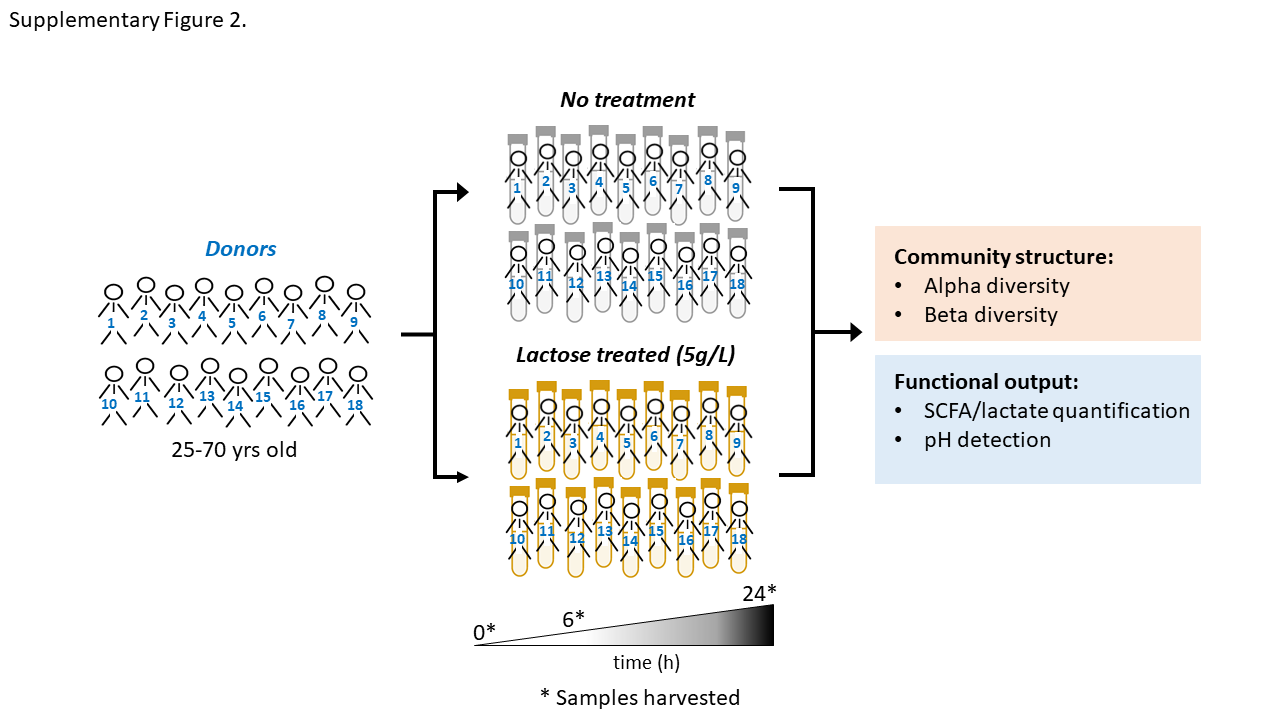

Supplement: Supplementary Figure 2 — Schematic illustrating the experimental design. *Samples harvested. [file Image_2.TIF]
